# Electroencephalogram (EEG) assessment of brain activity before and after electrical stunning in the Nile crocodile (*Crocodylus niloticus*)

**DOI:** 10.1038/s41598-023-47696-3

**Published:** 2023-11-20

**Authors:** K. J. Du Plooy, G. E. Swan, J. G. Myburgh, G. E. Zeiler

**Affiliations:** 1 Faculty of Veterinary Science, University of Pretoria, Private Bag X04, Onderstepoort, 0110 South Africa; 2 Exotic Leather Research Centre, University of Pretoria, Private Bag X04, Onderstepoort, 0110 South Africa; 3 Section of Anaesthesia and Critical Care, Valley Farm Animal Hospital, Pretoria, Gauteng South Africa

**Keywords:** Biophysics, Biotechnology, Ecology, Neuroscience, Physiology, Systems biology, Zoology, Environmental social sciences, Medical research, Neurology

## Abstract

Electrical stunning is used to capture crocodiles to perform routine management procedures. It is essential from a welfare point that electrical stunning must cause unconsciousness in animals. However, there is no information of whether or not electrical stunning causes unconsciousness in the Nile crocodile (*Crocodylus niloticus*). The purpose of the study was to assess brain activity before and after electrical stunning in crocodiles using a 5-channel referential electroencephalogram analysis to determine consciousness. Behavioural indicators and electroencephalogram recordings of 15 captive-bred crocodiles were captured and analysed using power spectral density analysis immediately before and after stunning and then at 60 s intervals until 5 min post-stunning. A standardised stun of 170 Volts was applied for 5–7 s on the wetted neck. Unconsciousness was defined as a decrease in alpha wave power and increase in delta wave power. Three of the electroencephalograms could not be assessed. Unconsciousness was identified in 6 out of 12 crocodiles and lasted for an average for 120 s. An increase in electroencephalogram waveform amplitude and tonic–clonic seizure-like waveform activity and behaviour indicators were not reliable indicators of unconsciousness. Further research should be focused on improving the efficiency and reliability of electrical stunning.

## Introduction

In South Africa, the farming of the Nile crocodile (*Crocodylus niloticus*) has developed because of growing local and international demand for the production of their skin and to a less extent their meat. Electrical-stunning (e-stunning) is an efficient method to capture and restrain crocodilians and other farm animals for the purpose of their handling and conduction of specific management procedures^1–6^. E-stunning needs to render an animal unconscious to enable performing any painful management procedure, or for the killing of a crocodile by means of decapitation, or spinal cord severance followed by pithing of the brain during slaughter^3,4,7^. The European Union council regulation (No. 1099/2009) criteria for the protection of animals at the time of killing additionally require electroencephalogram confirmation of the efficacy of e-stunning in animals to cause unconsciousness^8^. Despite the wide use of e-stunning on commercial crocodile farms no such electroencephalogram confirmation has yet been published in Nile crocodile. Criteria to indicate a suspicion that a crocodile is conscious include a pupillary light response, response to moving objects, eye movement, spontaneous blinking, intentional defensive responses to handling and tongue movements. In the absence of all of these criteria, then unconsciousness can be inferred^5^.

Measuring brain activity through electroencephalogram wave patterns is used to illustrate the state of consciousness^9,10^. There are four types of waveforms of the electroencephalogram tracing that can be associated with different levels of consciousness which are: (1) active; (2) transitional; (3) unconscious; and (4) iso-electric (flat line pattern)^10^. These waveforms can be used to evaluate the effectiveness of e-stunning. Frequency (Hz), amplitude (μV) and power (μV^2^) are all measurable constituents of an electroencephalogram recording and are used together to represent the amount of brain activity and allow meaningful interpretations of the recording when using frequency domain methods of analysis. The raw electroencephalogram tracing must undergo fast Fourier transformation before a frequency domain method of analysis can be applied. Fourier transformation separates the raw electroencephalogram tracing into four different wave types that oscillate within four different frequency bands: delta (δ; 0–4 Hz), theta (θ; 4–8 Hz), alpha (α; 8–12 Hz) and beta (β; 12–30 Hz) waves^10–12^. In humans, deeper stages of sleep, anaesthesia, and reduced consciousness are associated with more activity (power) of the slower waves (delta and theta) compared to the faster waves (alpha and beta) when analysed using a power spectral density (PSD) analysis (a frequency domain method of analysis)^10,11^.

Brain activity in production animal studies, where e-stunning was used to render the animal unconscious before killing, has been investigated using a bipolar montage for electroencephalogram recording and observing behavioural indicators of loss of consciousness^6,13,14^. The electroencephalogram recording and behaviour indicators assist in differentiating between effective e-stunning (achieving an unconscious state) and electro-immobilization (animal remains conscious but immobile). These studies confirm that there is more activity in the slower waves compared to the faster waves based on PSD analysis that indicated a state of unconsciousness^10^. We speculate that this outcome will be similar in crocodiles, however, their behavioural indicators may not be as easy to detect as in mammals, because of their physiological and behaviour peculiarities. A 5-channel referential montage can be used instead of a bipolar montage in order to obtain high quality electroencephalogram recordings. This method of electroencephalogram acquisition has not been previously described for crocodiles; therefore, an anatomical research component was required to determine the position and size of the brain, and the skull thickness to identify the best sites to place surface electrodes.

The aim of this study was to record brain wave activity and behaviour indicators pre- and post- e-stunning by means of a 5-channel referential electroencephalogram tracing to measure the state of coconsciousness in Nile crocodiles using a standardised crocodile e-stunner setup.

## Results

### Identification of surface electrode sites

**Figure 1:**
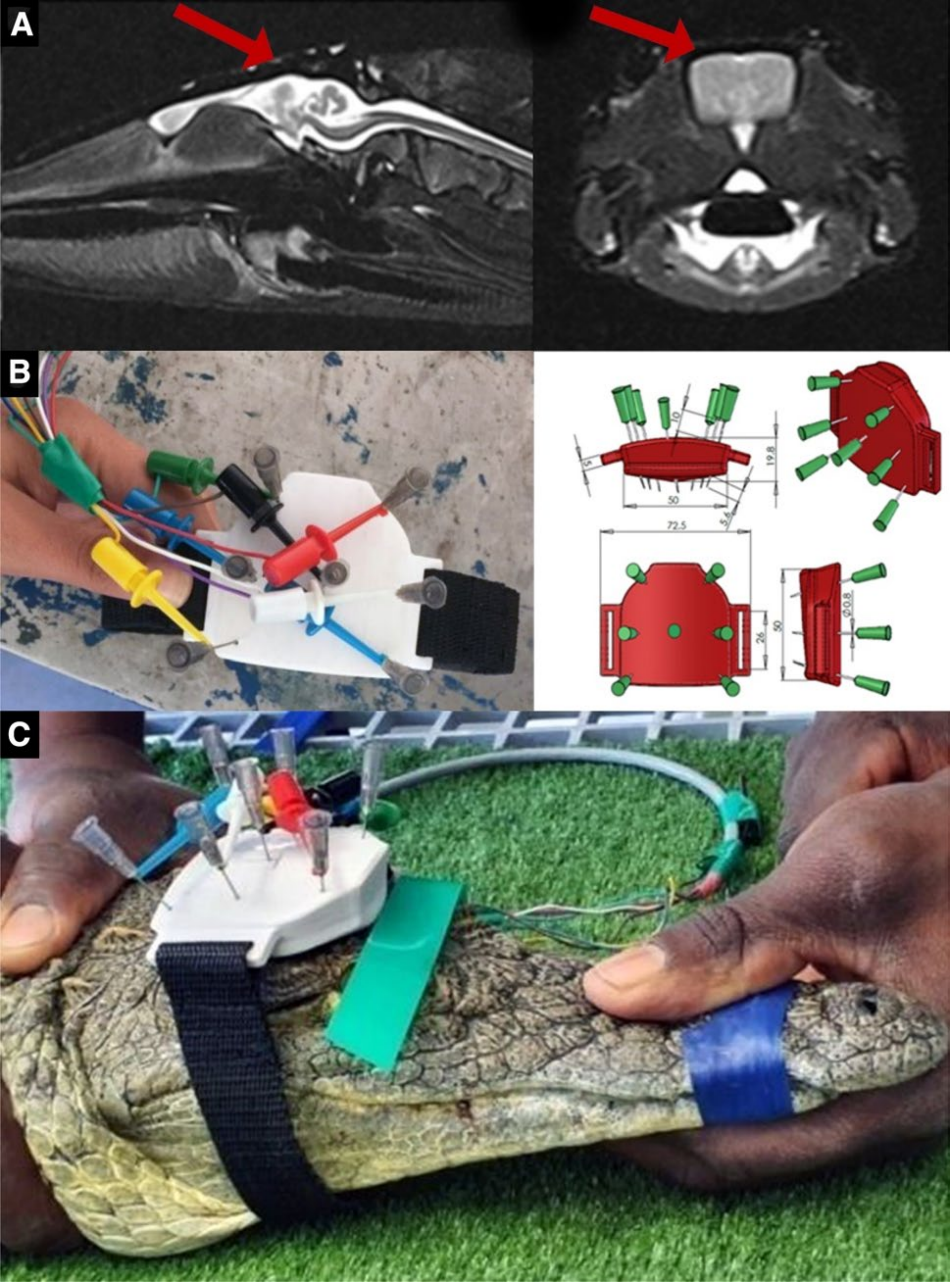
A series of figures depicting the stages of development of a custom 3-dimensional printed electrode placement skull-cap for continuous electroencephalography recordings. Midline sagittal and coronal magnetic resonance imaging (MRI) views of the brain parenchyma (white and light grey structures) within the cranial vault (dark grey) of a head specimen of a Nile crocodile (*Crocodylus niloticus*) illustrating that the best site to place the skull-cap was on the cranial plate (red arrows) (**A**). The skull-cap had 7 electroencephalogram electrode placement sites where the neutral and reference electrodes were the two rostral needles (**B**). The remaining 5 electroencephalogram electrode placements sites were for the electroencephalogram channels 1–5. A live crocodile with the electrode placement skull-cap placed on the cranial plate (**C**).

Seven electroencephalogram electrode placement sites were identified on the cranial plate based on magnetic resonance imaging study and dissections (Fig. 1). The number of electrodes used was limited by the size of the cranial plate. A 7-hole plastic skull-cap was custom made by 3-dimensional printing to fit similar sized crocodiles that was used to hold the electrodes in a fixed position during the electroencephalogram recording procedure.

### EEG Recordings and behavioural indicators

**Figure 2:**
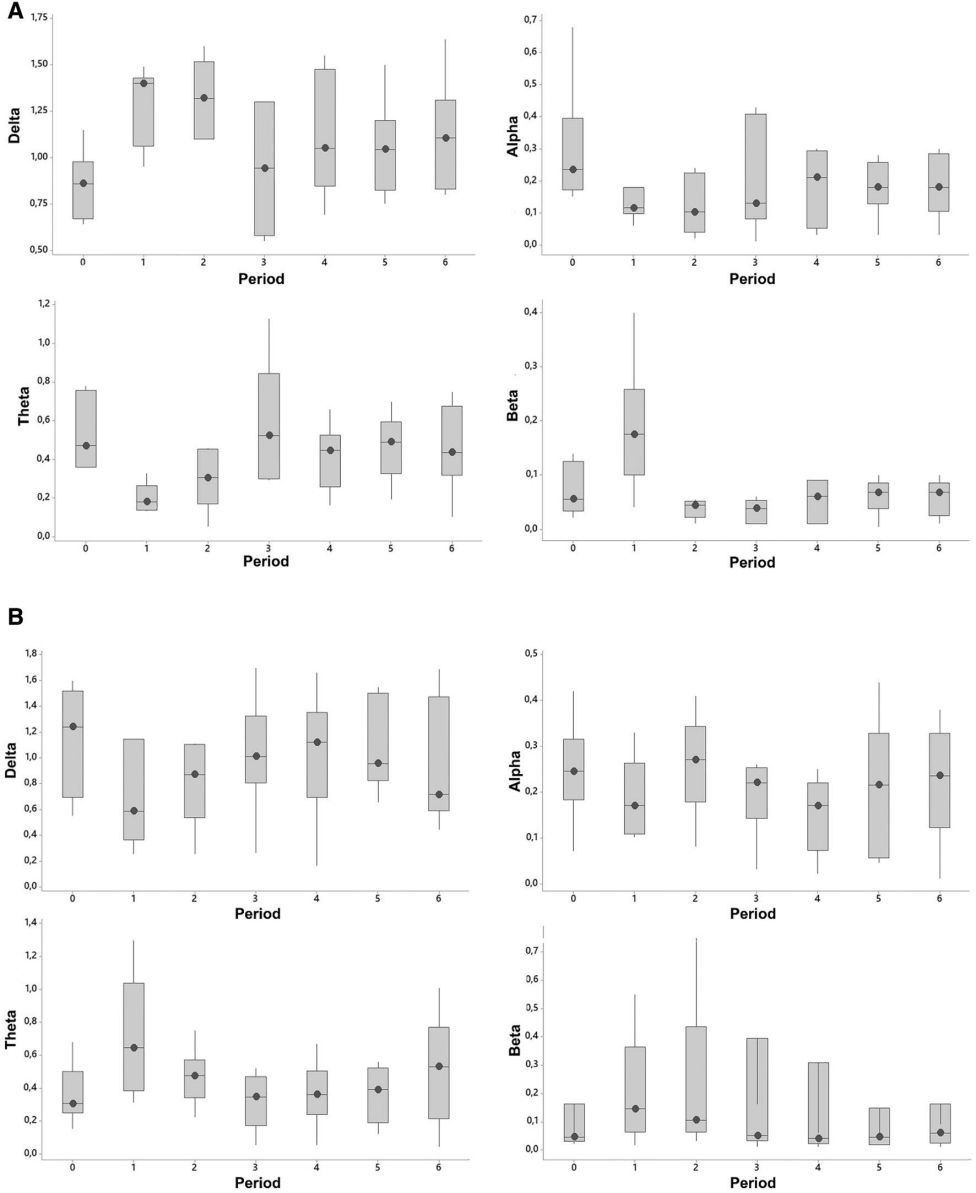
Power Spectral Density (PSD: μV^2^/Hz) for each frequency band (delta, theta, alpha and beta) before (period 0) and after (period 1–6) e-stunning of Nile crocodile (*Crocodylus niloticus*) that were effectively stunned (**A**) and not effectively stunned (**B**).

**Figure 3:**
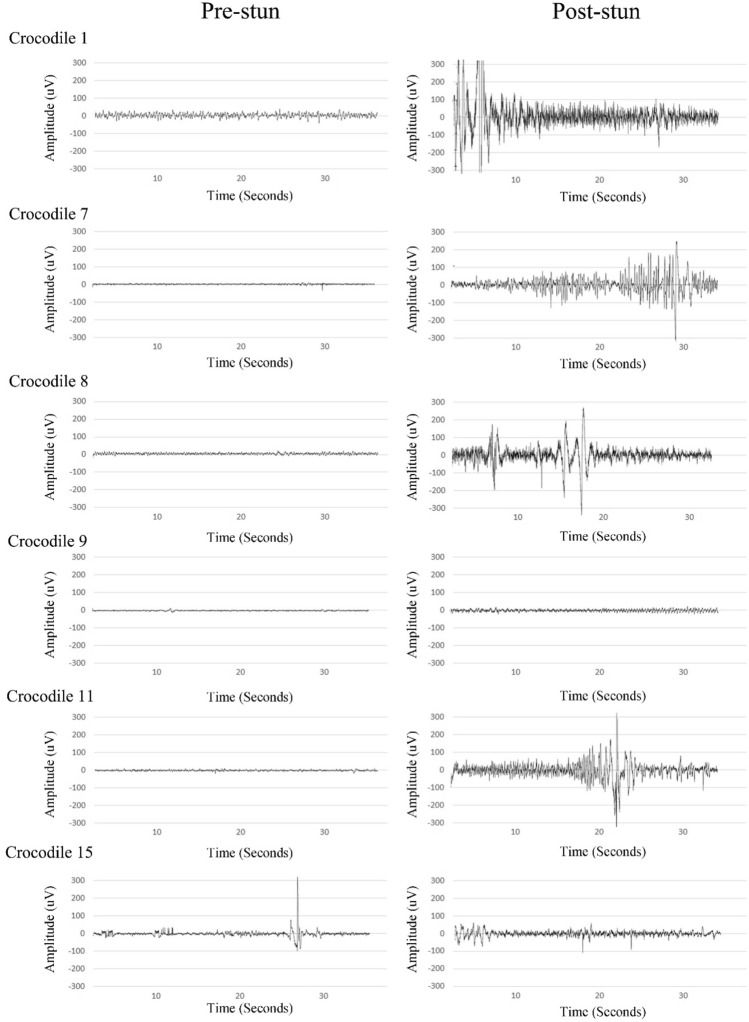
Raw electroencephalography tracings before and after e-stunning of Nile crocodiles (*Crocodylus niloticus*) that were effectively stunned.

**Figure 4:**
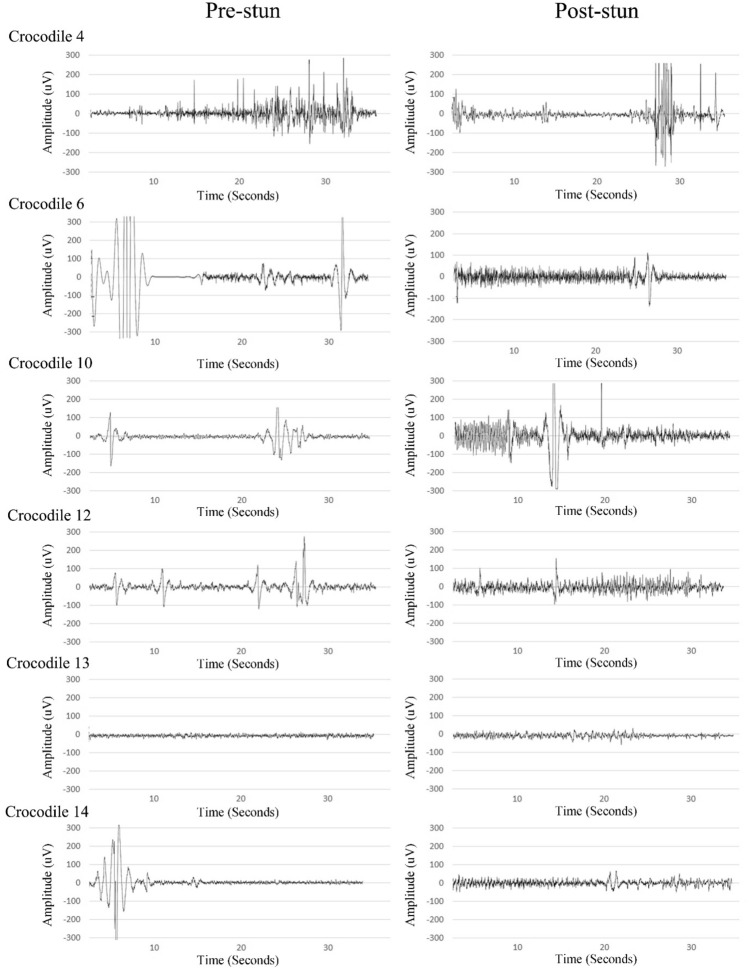
Raw electroencephalography tracings before and after e-stunning of Nile crocodiles (*Crocodylus niloticus*) that were not effectively stunned.

A state of unconsciousness (effective stun) was identified by a simultaneous increase in delta activity and a decrease in alpha activity and was demonstrated in 6 out of 12 crocodiles. Three of the electroencephalogram recordings could not be analysed at the pre-planned epochs likely because of poor electrode contact. This occurred in crocodiles 2, 3 and 5. Crocodiles 2 and 3 were stunned for 5 s and crocodile 5 for 7 s. In the effectively stunned crocodiles, the delta (*p* = 0.010) and beta (*p* = 0.045) power significantly increased, and theta (*p* = 0.004) and alpha (*p* = 0.015) power significantly decreased immediately after stunning (period 0–1) (Fig. 2A). Furthermore, the delta power returned to pre-stun values by time period 3, which is 120 s after completion of the stun. Whereas the alpha power returned to pre-stun values at time period 4, which is 180 s after completion of the stun. The remaining 6 crocodiles were not effectively stunned and considered to be in a state of electro-immobilization. In crocodiles that were not effectively stunned the delta power significantly decreased (*p* = 0.037) immediately after stunning (period 0–1) whereas theta (*p* = 0.055), alpha (*p* = 0.336) and beta (*p* = 0.20) power showed no significant change (period 0–1), immediately after stunning (Fig. 2B). The 40-s pre- and post-stun electroencephalogram waveforms were visually different between the crocodiles that were effectively (Fig. 3) stunned compared to those that were not effectively stunned (Fig. 4). The crocodiles that were not effectively stunned had more movement artefacts during the 40-s pre-stun recording because of escape attempts which was confirmed on the video recording. The post-stun recordings also demonstrated an increase in amplitude compared to the pre-stun recordings in all crocodiles.

Identifying behavioural indicators of stunned crocodiles was inconclusive because there were no behavioural observations specific to crocodiles effectively or not effectively stunned (Table 1). The crocodiles were mostly docile and showed little to no signs of distress after being captured manually and during pre-stun electroencephalogram recordings, except of short bouts of head and limb movements in 5 of the crocodiles that were not effectively stunned. Thus, overall, the crocodile demeanour made identifying behavioural indicators of unconsciousness unreliable. There were no apparent adverse effects observed in crocodiles resulting from e-stunning.

**Table 1:** Behavioural observations observed during and after e-stunning of Nile crocodiles (*Crocodylus niloticus*).

During	Post-stunning		
e-stunning	0–1 min	1–2 min	> 2 min
(12/12) arched back	(12/12) hind legs stiffened	(7/12) legs relaxed	*(1/12) 3 min blinked and flicked ears flaps*
(12/12) eyes closed	**(5/12) muscle contractions legs “walking motion”**	**(5/12) head lowered**	*(1/12) 3 min subtle tail movements*
(12/12) forearms extended initially	(2/12) eyes open	*(1/12) eyes open*	**(1/12) 3 min head down, toes relaxed**
(12/12) tail twitched	*(2/12) increase tail twitch*	(2/12) hind legs contracted then relaxed	**(1/12) 3 min body very still, eye movements**
*(5/12) limbs along body*	(2/12) tail ‘wagged’	**(2/12) tail twitch stop**	*(1/12) 3 min toe twitched*
(3/12) forearms stiffened alongside body	**(2/12) head partially lowered**	**(1/12) blinked**	**(1/12) 3 min head partially elevated**
(11/12) head up	**(1/12) toes twitched**	**(1/12) tail twitched**	**(2/12) 3–4 min no pedal reflexed**
*(2/12) forearms along body, hind legs extended*	**(1/12) subtle hind leg movements**	*(1/12) back relaxed flat*	*(1/12) 3–4 min reacted to touch and movements but 5 min no pedal reflex*
**(2/12) body twitched**	**(1/12) forearms relaxed**	*(1/12) toes splayed*	**(2/12) 4 min eyes relaxed**
*(2/12) toe twitched*	**(1/12) reacted to touch**	**(1/12) few deep breathes**	**(1/12) 4 min shallow breaths**
*(2/12) hind leg muscle tremors*	**(1/12) eye pupils constricted**	*(1/12) body twitched*	*(1/12) 5 min shallow breaths*
**(1/12) rapid tail twitch**	**(1/12) tail thrashed**	*(1/12) neck twitched*	**(1/12) 6 min aware of surroundings**
**(1/12) tail thrashed**		**(1/12) forearms along body**	(8/12) 5–7 min few deep breaths then normal breathing
**(1/12) head never lifted**		**(1/12) random body contractions**	**(2/12) 7 min hissed when picked up**
**(1/12) eyes half open**			**(1/12)7 min pedal reflex**
**(1/12) hind legs stiffened along body then extended**			*(1/12)* > *7 min head slightly elevated*
			**(1/12) 8 min blinked**
			**(1/12) 8 min no pedal reflex**
			*(1/12) 8 min shallow breaths*
			*(1/12) 8 min no pedal reflex but thrashed when picked up*
			**(1/12) 10 min vigorous movements when picked up**

(x/12) = number of crocodiles observed /a total of 12 crocodiles. Behavioural observations of effectively stunned crocodiles are in bold emphasis and not effectively stunned crocodiles are in italic emphasis and observations seen in both are in white.

### E-stunning data between effectively and not effectively stunned crocodile

The mean volts, AUC (amp × seconds) and amp-max delivered to all crocodiles in the current study were 171.40 ± 1.75 V, 5.48 ± 1.44 AUC, 1.09 ± 0.34 amp-max, respectively, and not different between effectively and not effectively stunned crocodiles. There were no significant differences in the total length, head length and body mass between the effectively stunned and not effectively stunned crocodile.

## Discussion

Reliable electroencephalogram tracings were obtained using a referential electroencephalogram recording. A state of unconsciousness was identified through electroencephalogram analysis. E-stunning of Nile crocodiles on the dorsal surface of the neck immediately behind the occipital bone, set at 170 V for 5–7 s, achieved variable results, with only half of the study animals showing electroencephalogram indications of unconsciousness despite all of the study animals receiving a stable and standardised electrical stun.

Power spectral density analysis is a universal accepted method of analysing electroencephalogram recordings, especially with more powerful computers and large number of electrodes used^11^. In this study, seven electroencephalogram electrodes were used which included neutral and reference electrodes and five channels using a referential montage. Referential montages include more channels than bipolar montages which have been previously used in e-stunned animal studies^6^. Referential montages are advantageous, as the best raw electroencephalogram data can be selected for analysis. Bipolar montages use only two electrodes per one channel making it not possible to select raw data for analysis.

Electroencephalogram studies have been conducted to monitor the brain activity changes after e-stunning and during anaesthesia (unconsciousness) in different species. American alligators (*Alligator mississippiensis*) under anaesthesia, showed a decrease amongst all frequency bands (alpha, beta, delta, and theta waves)^3^. Whilst humans under surgical anaesthesia, the alpha waves decrease, and the delta and theta waves become predominate at deeper levels of anaesthesia^9^. Effects of e-stunning on the brain activity in cattle and sheep have been researched more often. The researchers observed that the amplitude of the post-stun raw electroencephalogram recordings increases and that there are often signs of tonic–clonic seizures activity^1,8,14^. Pre-stun electroencephalogram recordings (i.e., conscious state) results from these studies showed amplitude readings of 10–50 µV^1,8,14^. Whereas post-stun electroencephalogram indicators for unconsciousness was defined as an increase in amplitude (600 µV or at least an increase by fivefold), and/or brain wave patterns resembling a tonic-conic seizure immediately after e-stunning^1,8,14^.

The European Union regulations stipulate that a head-only e-stun for all species and an electrified warm water bath for poultry require a tonic–clonic wave pattern on the electroencephalogram recordings as a description of an effective stun^15^.

This study defined ‘unconsciousness’ by the change in power within the different frequency bands (an increase in delta waves and a decrease in alpha waves), as well as signs of tonic–clonic wave patterns in the raw electroencephalogram recordings. All crocodiles demonstrated an increase in waveform amplitude, but only 50% were considered to be effectively stunned and most had tonic–clonic seizure activity. However, these wave patterns were demonstrated after the delta and alpha waves had returned to pre-stun power. Therefore, we cannot confidently recommend an increase in post-stun wave amplitude or tonic–clonic seizure activity as a reliable indicator of unconsciousness in e-stunned Nile crocodile.

Behavioural indicators could give a better indication of immediate effect as the raw electroencephalogram tracing cannot be analysed immediately while the animal is being e-stunned. Behavioural signs of loss of consciousness following e-stunning have been identified in livestock and poultry. The behaviour of crocodiles immediately after e-stunned have been described for saltwater crocodiles (*Crocodylus porosus*) and Nile crocodiles^4,16^. Initiation of the stunned state is rapid and comprises an initial tonic phase, with rigor and tail twitching^4,16^. After this period, the stunned crocodile becomes relaxed with legs splayed backwards, parallel to the body^16^. Stunned animals remain immobilized for 5–10 min, but most individuals regain movement after about 3 min^4,16,17^. These behavioural indicators were similar to those observed in the present study. However, the period of unconsciousness, according to the study definition, only lasted for a maximum of 2 min despite the crocodiles remaining immobilized for at least 7 min after stunning. Therefore, the transition from an effective stun to a state of electro-immobilization was not obvious. The outcome of the present study indicates that painful procedures or killing of crocodiles after e-stunning should be done immediately after the stunning procedure to ensure that the crocodile is unconscious. Behavioural indicators of consciousness and unconsciousness warrant further investigation to identify reliable indicators that would confirm an unconscious state. Evaluating reflexes (such as toe-pinch, tail-pinch, cloaca-pinch) could help determine conscious responses to noxious stimulation and should be included in future e-stunning research on crocodiles of a similar size to those used in the present study.

There were notable limitations to this study. The sample size was small and could not be accurately calculated because of the scarcity of similar studies in reptiles. However, the information in this study will allow for more suitable sample size calculations to be made in future. Electroencephalograms of crocodiles 2 and 3 stunned for 5 s and crocodile 5 stunned for 7 s could not be interpreted, therefore comment on the duration of stunning and level of consciousness cannot be made. However, crocodile 1 was effectively stunned using 5 s and no concerns on the remaining crocodiles’ electroencephalograms suggest the research team refined the data collection procedures as the day progressed. The study proposal set out the standardised setting for the e-stunner, however, at the start of data collection the stunning time variable was incorrectly set to 5 s which was noticed and corrected to 7 s before stunning the remaining 12 crocodiles. Because crocodile 1 was effectively stunned, we concluded that this change in stunning time did not impact the outcome of this study. We did not test other e-stunner electrical settings to determine if an increased current would cause an increase in e-stunning efficiency. Furthermore, although the crocodiles were doused with water before e-stunning, they were not submerged in water. However, in many farms, removing the crocodiles from water bodies and stunning after dousing with water is common practice to ensure the correct crocodile is stunned and to prevent any safety concerns such as drowning. Conversely, some farms adopt stunning while the crocodile is submerged in water, often in an isolation capture tank and our study did not emulate this e-stunning procedure. With the results of our study, future studies should compare these different stunning practices.

## Conclusion

The use of electroencephalography can be used to measure brain activity in Nile crocodiles, and it is a tool that can be used to determine changes in frequency band power. In this study unconsciousness was defined as an increase in delta and decrease in alpha frequency band power. An unconscious state was identified in 50% of the e-stunned crocodile that lasted for a maximum of 120 s. An increase in post-stun wave amplitude and presence of tonic–clonic seizure activity were not reliable indicators of unconsciousness. Furthermore, Nile crocodile demonstrate typical behavioural indicators of being stunned. However, these behaviour indicators were not reliable indicators of unconsciousness in the current study. E-stunning is an effective tool that can be used to capture crocodile safely and effective stunning results in a state of unconsciousness, further research should be focused on improving the efficiency and reliability of e-stunning.

## Methods

### Brain topography, skull thickness and electrode placement sites

Twenty-six head specimens were collected from captive-bred commercial crocodile farms after slaughter and were dissected along various coronal (*n* = 13) and sagittal (*n* = 13) plane cuts made by a bandsaw. Four of the heads underwent magnetic resonance imaging scans before dissection (Fig. 1). The scans were carried out using a 1.5 T machine (Ingenia MRI system; Phillips, South Africa) operated by experienced human radiologists. The skull progressively decreased in thickness from caudal (16.7 ± 1.2 mm) to rostral (4.5 ± 0.8 mm) over the brain cavity. The cerebral hemispheres of the brain were located ventral to the centre point of the cranial plate and this part of the brain was the largest portion that was closest to the surface.

### E-stunning procedure

#### Animals

Fifteen healthy crocodiles, 30–32 months of age, 144–160 cm in total body length were randomly selected by farm management from a group of growers that were housed in a closed communal pen. The crocodiles were captured manually and moved from the communal pen into an experimental house adjacent to the procedure room 2 days prior to the electroencephalogram recordings. A total of five crocodiles were placed in each of the three indoor concrete-floored pens that contained a water pond. The use of the live crocodiles in this study was approved by University of Pretoria Animal Ethics Committee (V084-18) and all study methods were performed in accordance with the relevant guidelines and regulations. The study is reported in accordance with ARRIVE guidelines. Once the data collection was complete, all crocodiles were returned to the owners who reintroduced them into the grower communal pens of the farm.

#### Procedure

A 7-hole plastic skull-cap that was custom made by 3-dimensional printing to fit similar sized crocodiles and strapped to the head of the crocodile was used to hold the electrodes in a fixed position during the electroencephalogram recording procedure. On the day of the electroencephalogram recordings, the skull-cap was assembled by placing injection needles (35 mm long; 21 gauge) through the 7 holes and 6 mm of the needle tip was bent to form a 90-degree angle which improved contact with the scale surface. On the crocodile end, the seven electroencephalogram cables were attached to the hub end of the needles using metal clips. On the electroencephalogram recorder end, the seven cables were attached to the mobile electroencephalogram recorder (Sleepwalker, Lifelines Ltd) and laptop (Inspiron, DELL). The seven cables were wrapped together to form an umbilicus and a quick-release connector was positioned midway along the 1.2-m-long cables to allow for rapid disconnection and reconnection.

Once the electroencephalogram recorder was setup the crocodiles were manually captured, one at a time, the snouts immediately taped and blindfolded and transferred to the procedure room. They were placed on a low work bench and physically restrained by two experienced animal caretakers, one holding the neck and the other the tail. The assembled skull-cap was placed onto the cranial plate of the crocodile and a 30 mm thick sponge was placed over the top to push the bent needles firmly against the scales. The entire assembly was secured using two self-adhesive material (Velcro) straps wrapped around the head. Then the raw tracing was visually inspected using compatible software (TrackIt version 2.8.0.12; Lifelines Ltd) for stability (not wandering off the isoelectric line or exhibiting excessive course oscillations around the isoelectric line) and an impedance check was done to ensure a value of less than 16 kΩ for each of the 5 electroencephalogram recoding channels. Once a stable tracing was obtained, the cloth blindfold was removed from the crocodile and a pre-stun electroencephalogram recording commenced for 5 min at a 200 Hz sampling rate. Following the pre-stun electroencephalogram recording, the cable umbilicus was disconnected, and the crocodile was prepared for e-stunning.

A research custom-built e-stunner (I’Vimbi technologies) that was powered by single phase mains electricity (220 V, 50 Hz) was used for this study. The e-stunner included a stunning controller referred to as the stunner and stunning apparatus referred to as stunning wand. The stunning wand was a hand-held plastic pole (1.4 m long) with a push-button at the operator-end and a V-shaped studded (three studs on each electrode) stunning electrodes at the crocodile-end of the wand. The volt-out controller of the Stunner was set at 170 V, and the timer set at 5 s stunning period for the first three crocodiles and 7 s for the remaining 12 crocodiles examined.

The data logger was set to record volts, amps and hertz applied for each crocodile and duration of stunning. For the stunning procedure, the neck and head region of the crocodile was doused with water and the stunning wand electrodes were pressed firmly at approximately 50° angle to the horizontal plane, on the dorsal surface of the neck immediately behind the occipital bone. The push-button was activated and the electrodes kept in the same position on the crocodile until the timer had run out. Immediately thereafter, the EEG cable umbilicus was reconnected, and the electroencephalogram recording continued for 7 min during the post-stun period. Throughout the procedure, for each crocodile, the principal investigator digitally video recorded the pre- and post-stunning periods and recorded behavioural indicators.

After completion of the post-stun, meloxicam (0.5 mL; Metacam 20 mg/mL; Boehringer, South Africa) was administered subcutaneously and the skull-cap assembly were removed, the crocodile was weighed and then returned to their experimental pen. The next crocodile was captured and brought to the procedure room. This same procedure was followed until all crocodiles were examined in 1 day. Crocodiles were returned to their communal pen the following day.

### Data analysis

#### Electroencephalogram recording data

Raw electroencephalogram recordings, consisting of five channels per recording, were uploaded to an open-source software (Brainstorm software package, freely available for download online under the GNU general public license at http://neuroimage.usc.edu/brainstorm) for processing and analysis. The raw electroencephalogram recordings were reviewed manually for signal artefacts and excessive movement away from the isoelectric line. A band-pass filter (1 Hz and 35 Hz) was applied to the raw electroencephalogram tracing and was reassessed visually to detect the three best performing channels out of the five. If at least three channels could not be identified because of poor signal quality, low amplitude (< 5 μV), excessive movement artefacts or excessive shifting away from the isoelectric line then the entire crocodile electroencephalogram recording was discarded and not analysed further nor included in the statistical analysis. Crocodiles 2, 3 and 5 were removed from the study because the electroencephalogram recordings could not be analysed.

A series of seven 4-s-long epochs were identified on stable, artefact free electroencephalogram recordings and marked, as follows: 1 pre-stun epoch (period 0); 1 post-stun epoch (period 1) identified within 1–7 s after reconnecting the cable umbilicus and 5 additional post-stun epochs (periods 2–6) exactly every 60 s after the start of the first post-stun epoch. First, each epoch was visually inspected for stability and to ensure they were artefact free. Then the three-channel epoch was standardised by applying an average reference montage. The three-channel epochs were then analysed using the Welch method of power spectral density analysis (time window: 4 s; Hamming window length: 0.5 s; window overlap ratio: 50%; decomposition into delta [2–4 Hz], theta [5–7 Hz], alpha [8–14 Hz] and beta [15–30 Hz] frequency bands). The three spectrum graphs were normalised and then averaged using root mean square averaging. The final averaged PSD analysis was used to obtain power values (μV^2^/Hz) for each frequency band of interest for each epoch^12^. The total power was calculated by adding all frequency band powers. The percentage of power within each frequency band was calculated by dividing the power within the band by the total calculated power and converted to a percentage. Additionally, 40 s epochs were extracted from the filtered electroencephalogram recording during the pre-stun (40 s before disconnecting electrode cable umbilicus) and post-stun (40 s after reconnecting the electrode cable umbilicus) periods for visual comparisons and to identify tonic–clonic seizure activity.

The PSD data were assessed for normality through plotting of histograms, inspecting descriptive analysis and the Anderson–Darling test for normality. The pre-stun power within each frequency band was compared to post-stun power using Kruskal–Wallis test. Crocodiles with electroencephalogram data that demonstrated a post-stun increase in delta power and a decrease in alpha power were considered unconscious and therefore an effectively stunned. Crocodile with an absence of these electroencephalogram findings were considered to be conscious but electro-immobilized and therefore not effectively stunned. The PSD analyses were compared and matched descriptively to their behavioural indicators that were recorded and interpreted with the aid of reviewing the digital video recordings. Data was analysed using commercially available software (MiniTab 18.1; MiniTab Inc) and significances interpreted at *p* < 0.05.

### E-stunner data

The data recorded by the stunner data logger was downloaded to a laptop (Inspiron, DELL) for analysis. The volts, amps and hertz were captured per second for the duration of the stun for each crocodile. The area under the curve (AUC) for amps versus time was calculated using the Trapezoidal method (AUC_1−n_ = ∑{$(\frac{Cp1+Cp2}{2})(t2-t1)\}+\{(\frac{Cp2+Cp3}{2})(t3-t2)\}+\dots$) (1) where Cp is the amps and t is time (seconds). Normality and homogeneity of variance of the stunner data was assessed using the respective Shapiro-Wilks and Levene’s tests. The demographic data (total length, head length and weight) of the crocodiles and stunner data of crocodiles were compared between effectively stunned and not effectively stunned crocodile using Student’s t-tests. Statistical analyses were performed using IBM SPSS v26 (IBM Corp, USA).

## Data Availability

The datasets generated during and/or analysed during the current study are available from the corresponding author on reasonable request.

## References

[1] Cook CJ, Devine CE, Gilbert KV, Maasland SA, Blackmore DK (1996). Changes in the release of amino acid neurotransmitters in the brains of calves and sheep after head-only electrical stunning and throat cutting. Res. Vet. Sci..

[2] Ferree TC, Luu P, Russell GS, Tucker DM (2001). Scalp electrode impedance, infection risk, and EEG data quality. Clin. Neurophys..

[3] Nevarez JG, Strain GM, Da Cunha AF, Beaufrere H (2014). Evaluation of four methods for inducing death during slaughter of American alligators (*Alligator mississippiensis*). Am. J. Vet. Res..

[4] NRMMC (Natural Resource Management Ministerial Council). Code of practice for the humane treatment of wild and farmed Australian crocodiles. NRMMC: Canberra, Australia (2009).

[5] World Organisation for Animal Health, formerly the Office International des Epizooties (OIE). Killing of reptiles for their skins, meat and other products. OIE Terrestrial Animal Health Code, 28^th^ Edition, Volume 1, Chapter 7.14.www.o.i.e.int/standard-setting/terrestrial-code. (2019).

[6] Verhoeven MTW, Gerritzen MA, Hellebrekers LJ, Kemp B (2015). Indicators used in livestock to assess unconsciousness after stunning: A review. Anim..

[7] Davis KA, Devries SP, Krieger A, Mihaylova T, Minecan D, Litt B, Wagenaar JB, Stacey WC (2018). The effect of increased intracranial EEG sampling rates in clinical practice. Clin. Neurophys..

[8] EFSA (2004). Opinion of the scientific panel on animal health and welfare on a request from the commission related to welfare aspects of the main systems of stunning and killing the main commercial species of animals. EFSA J..

[9] Harighira S (2015). Changes in the electroencephalogram during anaesthesia and their physiological basis. Br. J. Anaesth..

[10] Tadel F, Baillet S, Mosher JC, Pantazis D, Leahy RM (2011). Brainstorm: A user-friendly application for MEG/EEG analysis. Comput. Intell. Neurosci..

[11] Levy MD, Warren J (1987). Effect of epoch length on power spectrum analysis of the EEG. Anesthes..

[12] Pfitzer S, Ganswindt A, Fosgate GT, Botha PJ, Myburgh JG (2014). Capture of farmed Nile crocodiles (*Crocodylus niloticus*): Comparison of physiological parameters after manual capture and after capture with electrical stunning. Vet. Rec..

[13] Grandin, T. Electric stunning of pigs and sheep [Online] https://www.grandin.com/humane/elec.stun.html: Colorado State University (2015). Accessed 2019.

[14] Sánchez-Barrera IC, Albarracin W, Rojas MJ (2014). Electroencephalographic spectrum power of sheep’s brain after stunning. J. Appl. Anim. Res..

[15] European Union (2009). EU regulations no 1099/2009 of 24 September 2009. Off. J. Eur. Union.

[16] CFAZ (Crocodile Farmers Association of Zimbabwe). Codes of practice. CFAZ: Harare, Zimbabwe (2012).

[17] Franklin CE, Davis BM, Peucker S, Stephenson H, Mayer R, Whittier J, Lever J, Grigg G (2003). Comparison of stress induced by manual restraint and immobilisation in the estuarine crocodile (*Crocodylus porosus)*. J. Exp. Zool. Part A Comp. Experimen. Biol..

